# The Use Of Fundus Photography In The Emergency Room—A Review

**DOI:** 10.1007/s11910-025-01417-7

**Published:** 2025-04-11

**Authors:** Samuel D. Browning, Julia M. Costello, Hamish P. Dunn, Clare L. Fraser

**Affiliations:** 1 Faculty of Medicine and Health, The University of Sydney, New South Wales, Australia; 2 Save Sight Institute, The University of Sydney, New South Wales, Australia; 3 Faculty of Medicine, The University of New South Wales, New South Wales, Australia; 4 Port Macquarie Eye Centre, Port Macquarie, New South Wales, Australia; 5 Port Macquarie Base Hospital, Port Macquarie, New South Wales, Australia

**Keywords:** Non-mydriatic fundus photography (NMFP), Emergency physician (EP), Emergency room (ER), Direct ophthalmoscopy (DO), Diabetic retinopathy (DR), Ocular fundus (OF)

## Abstract

**Purpose of Review:**

The ocular fundus reveals a wealth of pathophysiological findings which should change patient management in the emergency room (ER). Traditional fundoscopy has been technically challenging and diagnostically inaccurate, but technological advances in non-mydriatic fundus photography (NMFP) have facilitated clinically meaningful fundoscopy. This review presents an update on the literature regarding NMFP and its application to the ER, illustrating pivotal publications and recent advances within this field.

**Recent Findings:**

NMFP’s application in the ER is demonstrably feasible and seamlessly integrates into emergency physicians’ (EP) diagnostic workflows in a clinically meaningful and time efficient manner. The images of the ocular fundus (OF) generated by NMFP are consistently high quality, allowing a greater diagnostic accuracy to EP and ophthalmology interpreters alike. Digital NMFP images facilitate effective ophthalmology input via telemedicine to review the images in the ER. NMFP has been shown to change management decisions in the ER, improving patient and departmental outcomes. Interpretation of fundus images remains a medical education challenge, and early research highlights the potential for artificial intelligence (AI) image systems of NMFP to augment image interpretation in the ER.

**Summary:**

NMFP can change the ER approach to OF assessment, however the factors limiting its routine implementation need further consideration. There is potential for AI to contribute to NMFP image screening systems to augment EPs diagnostic accuracy.

## Introduction

Since the 1800s, examination of the ocular fundus (OF) has largely been conducted with the aid of the direct ophthalmoscope. This technology has remained relatively unchanged since its inception, offering minimal improvement to the view of the OF since its first iterations [1]. Within the field of ophthalmology the OF assessment has evolved to incorporate a range of modern investigational modalities [2], however in the non-ophthalmic setting direct ophthalmoscopy (DO) is still the most prevalent examination tool available [3]. DO presents many barriers to use for non-experts, such as the restricted 5-degree view of the retina [4] that is both technically and practically challenging to conduct, interpret, teach, and assess inter-rater reliability [5]. Non-ophthalmic physicians have low-levels of confidence in interpreting fundoscopic signs [6–8], ultimately limiting DO’s diagnostic value [9–11]. For these reasons, the examination of the OF has been effectively removed from the non-ophthalmic physicians repertoire of patient assessment skills [12].

The OF is a critical assessment to complete a comprehensive physical examination across many disciplines where a range of systemic inflammatory [13], medical, and neurological [5, 14] pathology can be manifest, often heralding more serious disease or progression of illness [15]. This is particularly relevant in the emergency room (ER) setting, where early and accurate detection of disease entities guides ER work up, improves quality of care, and facilitates timely management [16]. Fortunately, recent technological advances with the development of non-mydriatic fundus photography (NMFP) in the last decade have been shown to offer a superior alternative to DO in many non-ophthalmic settings [9] addressing many of these hurdles. The use of NMFP in the ER has the potential to shift the paradigm of OF examination and return fundoscopy to a valuable component of clinical assessment, simultaneously improving patient outcomes and departmental flow.

This review provides an update on the use of fundus photography in the ER setting, emphasising recently published literature whilst acknowledging the important context provided by landmark publications and highlighting the potential for improved patient outcomes.

## Foundational Studies: Establishing NMFP in the ER

Landmark studies which first showed that NMFP was an optimal technique for OF examination in the ER continue to serve as guiding principles for evaluating the data, analysing use, and shaping current understanding of NMFP in this setting. These pivotal studies provide an ongoing valuable framework for assessing the feasibility, effectiveness, and potential impact of this relatively new field in the world of Emergency Medicine.

The FOTO-ED studies in particular have been instrumental in demonstrating the practicality and benefits of NMFP in ERs. These landmark studies were conducted in Atlanta, Georgia (USA). ‘FOTO-ED Phase I’ was the first of a three-phase series of studies directed at determining the feasibility of NMFP use in the ER [12]. This phase enrolled patients presenting to an adult ER with inclusion criteria of headache, acute visual change, neurological deficit, or diastolic blood pressure > 120 mmHg. NMFP images were taken in 350 eligible patients by a nurse practitioner trained in use of the camera, with 83% of enrolled patients having at least one diagnostic image, and only 3% had no images of any diagnostic value. Despite emergency physicians (EP) being aware of the audit, just 14% of these patients had ocular assessment with DO performed as routine clinical care, demonstrating just how underutilised OF assessment is in the ER. This study highlighted several critical features of NMFP which contribute to its feasibility in the ER setting. Notably, NMFP provides a 45-degree wide view of the retina without needing to dilate the pupils pharmacologically, and is a relatively low-cost piece of equipment [17]. NMFP can be performed by non-physician staff members with minimal training [12], reducing the burden on constrained physician/ER doctor resources. The median image capture time per patient was 1.9 min, and was highly acceptable for patients. Of the enrolled patients 44/350 (13%) had relevant OF pathology, only 6 (2%) had been detected by clinical DO.

The FOTO-ED studies came in the setting of several law-suits where serious OF pathology was missed because the physicians had not done fundoscopy [18]. Therefore, the second phase of the FOTO-ED studies was directed at determining the diagnostic accuracy and use of NMFP by ER physicians. This study [9] enrolled patients with the same inclusion criteria, and captured images according to the same protocol as FOTO ED I. NMFP images were then immediately placed in the patient’s electronic medical record, and EPs were alerted to completed case report forms for their patients. 354 patients were enrolled, of which 35 (10%) had relevant pathology. Compared to the reference standard of ophthalmologist review of NMFP images, EPs had a 46% sensitivity and 96% specificity. By comparison EPs use/non-use of direct ophthalmoscopy had 0% sensitivity for fundus pathology. Whilst demonstrating quite marked deficits in EP detection and interpretation of OF pathology, the use of NMFP was shown to significantly aid EPs diagnostic acumen without any further training. More than one-third of the images were deemed useful by EPs, either in identifying abnormalities or excluding other relevant pathology.

The third phase of the FOTO-ED study [19] aimed to address limitations identified in the earlier phases, particularly the need for improved interpretation skills among EPs. Implementing web-based training programs, the study involved 587 patients but yielded disappointing results. There was no significant change in NMFP review rates, with EPs reviewing 45% of patients' images before training and 43% after. Surprisingly, the ability to correctly identify abnormalities slightly decreased from 67 to 57% post-training, and while identification of normal cases marginally improved from 80 to 84%, this was not statistically significant. The study's outcomes uncovered that there was not an easy fix to improving non-expert’s ability to interpret NMFP which posed a challenge to the effective implementation of NMFP in ER settings. This unresolved challenge points to the need for innovative training methods or systemic changes to bridge the gap between the potential of NMFP and its practical application in emergency medicine.

## NMFP Technologies: A Comparative Review

**Fig. 1:**
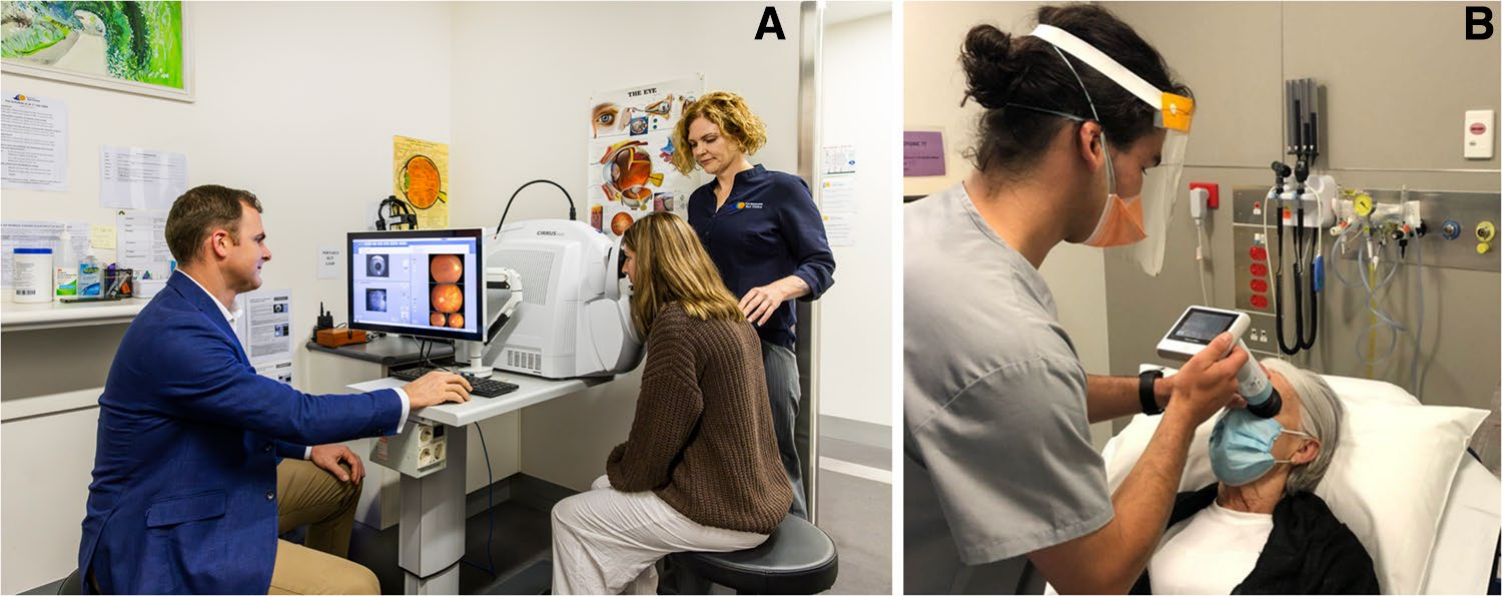
**A** Conventional fundus photography in clinical practice, and; **B** Portable non-mydriatic fundus photography at the patient bedside in an emergency department

The range and number of NMFP technologies has significantly expanded in recent years with tabletop devices considered the gold-standard of NMFP [11]. The limitations of table-top devices include the requirement for unwell patients to mobilise to the assessment area as well as the significant expense in comparison to alternative handheld devices (see Fig. 1A) which allow for patients to be examined at the bedside (See Fig. 1B). Das et al [20] compared four different handheld NMFP devices with a tabletop NMFP device in both non-mydriatic and mydriatic conditions. They noted that the handheld device had excellent rates of image acquisition, the same as the table-top device (100%). Image quality, optic disc gradability, and vascular morphology gradability were comparable with, and in some cases superior to the tabletop device. Handheld NMFP has consistently captured high-quality images of diagnostic utility in a simple to operate, non-invasive and versatile manner when used in an ER setting.

Alternate NMFP technologies including smartphone fundoscopy and smartphone-integrated handheld devices, along with aforementioned handheld NMFP devices, provide similar advantages over table-top devices. A growing body of literature reviews the use of integrated smartphone-attachment NMFP technologies and their clinical applications. Lord et al [21] were the first to demonstrate how fundus images could be captured using smartphone attachments. This provided the basis for the majority of portable phone-attached NMFP technologies now in existence [22, 23].

While smartphone fundoscopy currently has a smaller field of view to NMFP, it shows promise as a valuable tool in overcoming the technical challenges of DO and reducing the barriers to learning and integrating fundoscopy in clinical practice. A randomised crossover trial of 146 medical students compared various fundoscopy modalities, including a tabletop non-mydriatic fundus camera; two types of direct fundoscopy; and three types of smartphone fundoscopy and found smartphone fundoscopy was perceived as more useful and easier to use than other modalities [24].

The FUNDUS study (Fundoscopy Use in Neurology Departments and the Utility of Smartphone Photography) was a prospective, randomised crossover study comparing the effectiveness of a handheld NMFP device and smartphone fundoscopy (SF) with standard care using traditional direct ophthalmoscopy (DO) for detecting fundus pathology in neurology inpatients [14]. Both devices showed superior performance compared to DO, which was rarely performed and missed all abnormalities. NMFP images generated slightly better diagnostic accuracy, with neuro-ophthalmologist review achieving 45% sensitivity for OF pathology. Comparatively, neuro-ophthalmologist grading of smartphone fundoscopy images achieved a sensitivity of 40%. These results suggest that both NMFP and SF outperformed DO, with NMFP having superior diagnostic performance over SF.

Hafiz et al [25] recently published their study of a new portable, handheld NMFP device designed for use in conjunction with smartphone technologies, allowing for wide-view OF images to be taken using a smartphone camera. Comparable smartphone based NMFP technology with video-based image capture capabilities, and inexpensive smartphone camera attachments have also been described [26]. A number of newer devices, namely the Remidio NM-10, are designed as handheld devices for bedside NMFP, but have smartphone connectivity for image capture [27]. There are also bench/desktop NMFP devices leveraging smartphone attachment technology to harness what is currently regarded as the gold standard in image capture capabilities [20]. These smartphone-integrated NMFP technologies have implications for use in telemedicine and applications in resource-limited situations and models of care, however further research validating this for real-world use is necessary [28].

It is important to note that the use of smartphone-based fundoscopy and other mobile health applications raises significant privacy concerns, as these devices can potentially collect and transmit sensitive patient data. In some health systems these devices are not approved for use for these reasons and at the very least their use necessitates robust security measures and transparent privacy policies to protect users' personal health information.

The advantages of the handheld devices have meant they are frequently the preferred devices utilised in published ER studies, and therefore predominate this review.

## NMFP in the ER: Assessing Feasibility & Clinical Value

ER workflow prioritises speed and efficacy [29], where patients are ideally triaged, assessed, and appropriately referred within a narrow time-frame [30]. EPs have to effectively manage their time across a diverse range of patient presentations, and apply rapid decision-making processes. It is in this context that NMFP offers advantages for improved ER fundus assessment.

A key investigation using cross-sectional analysis conducted in two Australian metropolitan ERs, implementing both desk-top and hand-held NMFP technologies [11]. This study prospectively enrolled 345 patients with presenting complaints warranting fundoscopic assessment. NMFP image quality was graded as ‘high’ in 89% of patients. Although there was no difference in the rate of image quality detected between tabletop and handheld NMFP devices, the handheld device was better utilised. The reported median image acquisition of just 2 min illustrating the efficiency of NMFP in the busy ER environment. These findings, along with further research undertaken in regional Australia [10–12, 31, 32] validated and extended the findings of the FOTO ED phase II [31] by clearly demonstrating how NMFP optimises ER decision-making processes, and facilitates a better use of healthcare resources. These results established NMFP as not simply a feasible and easily integrated, diagnostically superior alternative to DO in the ER, but as a screening tool capable of driving improved patient and departmental outcomes in this setting.

These findings align with other studies that have shown the feasibility and efficiency of NMFP. Teismann et al [32] enrolled 123 eligible ER patients, of which 93 (75.6%) had one or more diagnostically useful images with the application of handheld NMFP. Patients ranked their comfort to a mean score of 4.6/5 with the NMFP examination process, and mean elapsed time to complete photography was 109.6 s (~ 1 min 50 s). Despite its limited sample size, these findings were congruent with FOTO-ED phase I [12] and other studies [9, 10, 33, 34]. The ease-of-use in non-ideal settings, for instance in high ambient light and bedside examination of immobile or uncooperative patients, positions handheld NMFP for success in the chaotic ER environment (see Fig. 1). NMFP operation can be rapidly taught to non-physicians without a compromise on image quality [12, 35], making it more feasible to generate capacity within an ER department for implementation.

## NMPF and the Emergency Physician: Performance, Accuracy, & Interpretation

Despite fundoscopy potentially providing a useful diagnostic tool, without the confidence in their skills to examine the OF, EPs often resort to other more time-consuming investigations to exclude serious pathology. Teismann et al. observed EPs conducted DO of their own accord for 19 (15.4%) of the eligible patients enrolled in the study. Anecdotally, from the study by Dunn et al., EPs were found to be using the NMFP whilst on ER night-shift for 17/264 eligible patients, and never using DO [10]. It has been shown that ophthalmic skillsets decline without regular practice [7, 36], and the difficulties associated with DO are a clear impediment to maintaining OF interpretation skills. Indeed, when using DO, EPs have been shown to demonstrate sensitivity of 0% and specificity of 47% for OF pathology [10]. NMFP offers an easier way to examine the OF, and EPs are thus more likely to incorporate it in their patient work-up.

When using NMFP images, EPs had a sensitivity of 42% and specificity of 82% for detecting urgent pathology [11]. Other key studies document sensitivities ranging from 29–46%, and specificities between 82–96% [9, 10]. Of note, EPs were more prone to missing OF pathology (false negative rate of 71%) compared to over-diagnosing normal retina’s (16.5%), highlighting a particular deficit in detection of clinically relevant findings [10]. These studies establish both the diagnostic advantages of NMFP when utilised in the ER setting, yet the ongoing shortfalls in EPs’ OF interpretation skills.

Bruce et al. conducted a web-based in-service educational training program delivered to 14 EP care providers[19]. This brief 30-min educational tool did not show significant benefits in increasing EPs diagnostic acumen for either normal and abnormal ocular fundi (65% pre vs 71% post-training, p = 0.06), and there was no difference in diagnostic accuracy between trained and untrained EPs. However, it was shown that EPs trained in OF pathology detection reviewed a greater number of NMFP images in the clinical setting (45% vs 35%, p = 0.03). This suggests that increased exposure to NMFP images may naturally increase its clinical uptake, further research is required to better understand how to improve OF interpretation skills. Other studies have shown promise in using e-learning to improve fundus interpretation [37, 38] including an e-learning program which improved medical students' accuracy in interpreting patients’ optic discs in a randomised trial (mean improvement 4.5%: 95%CI 3.7–5.2%, p < 0.001) [39].

In the absence of further education, the direct crossover of DO to NMFP respectively showed that EPs’ detection of acute fundus pathology improved from 0 to 29% sensitivity, and 59% to 84%, validating the findings of the FOTO-ED studies [10–12, 31, 32].

## NMFP in the ER: Moving From Research to Routine Use

Despite the clear ER benefits of NMFP screening protocols, integration of NMFP screening protocols in ERs worldwide remains limited. Ensuring the uptake of NMFP by ER doctors and clinical care providers is important for improving patient outcomes, however there are limited studies investigating how NMFP can be better utilised and implemented by EPs. While supporting EPs diagnostic interpretation of NMFP images through teaching and education may itself drive technological uptake, there remains a significant gap between the demonstrable benefits of NMFP use within the ER, and its actual implementation and utilisation across ERs [8]. Qualitative research into fundoscopy, including in the ER, found that there were medical cultures of accepted incompetence in fundoscopy, misperceptions of senior clinician disapproval, and that education should focus at critical stages of training and practice to shift clinicians’ perceptions of the clinical utility or futility of fundoscopy [8].

Current and recent implementation studies have built upon the foundational work of landmark and key studies, exploring the challenges and strategies for integrating NMFP into ER workflows. Berman et al. recently published a quality improvement project utilising Kotter’s 8-Step Change Model to successfully implement a non-mydriatic fundus camera combined with optical coherence tomography (OCT). This approach resulted in sustained use, with 1,274 patients imaged over the course of a year, substantiating its viability as a diagnostic tool in a non-ophthalmic setting. This project was led by highly influential figures in this field, a level of leadership and expertise that is not universally available, thus representing a limitation to the generalisability of this implementation process. Additionally they used an embellished and expensive tabletop device, the drawbacks of which have been previously discussed. These limitations underscore the need for further exploration and in depth examination of implementation processes for NMFP [40].

The authors of this review have conducted unpublished mixed-methods investigations into the implementation of NMFP in the ER [41]. Preliminary analysis suggests key barriers to the uptake of NMFP include limited integration of NMFP images with electronic patient management systems, ER workflow habits and culture which bypass fundoscopy in patient work-up, low EP awareness of the utility of NMFP, and constantly evolving ER workforces with high staff-turnover. It was also apparent that EP’s confidence in interpreting normal and abnormal OF would naturally improve with more regular, high-quality exposure to the OF itself, which a fully-implemented NMFP screening protocol provides. Further research is required to identify ways to facilitate large-scale implementation of NMFP into ERs.

Despite the seminal work in the first phase of FOTO_ED illustrating the effectiveness of nurse practitioners in obtaining high-quality NMFP, much of the literature on education and implementation relating to this in ER remains focused on medical/physician led imaging and training [12]. Even implementation projects that claim to use multidisciplinary approaches appear to have a doctor-centric perspective [40], overlooking the potential for broader nursing involvement in assessment and care delivery as is seen in the use of NMFP in the diabetes space [42].

## Improving Patient outcomes: The Role of NMFP in Identifying Pathology & Impact on Patient Management

Excluding ‘red-flag’ or life-threatening pathology is imperative in ER patient assessment and management. This depends on the ability to rapidly detect critical illness, whilst also excluding important differential diagnoses to appropriately guide investigations and management. In validation of the FOTO-ED studies multiple studies mentioned in this review reinforce the clinically significant proportion of fundus pathology found in 10–16% of relevant ER presentations [9–12, 32], underlining the relevance of improved ocular assessment and screening protocols. Manifestations of ophthalmic and systemic conditions found are listed in Table 1.

**Table 1:** Fundoscopic manifestations of ophthalmic & systemic diseases and their clinical implications

Systemic Disease/Condition	Retinal Fundoscopy Findings	Potential Clinical Outcomes if Missed
Diabetes	Microaneurysms, dot and blot haemorrhages (Hb), hard exudates, cotton wool spots (CWS), neovascularization	Progressive vision loss, blindness; increased risk of systemic complications (e.g., renal failure, cardiovascular events)
Hypertension	Arteriolar narrowing, arteriovenous nicking, flame-shaped Hbs, Disc oedema (in severe cases)	Stroke, heart failure, kidney damage, accelerated hypertension (malignant hypertension), increased risk of cardiovascular events
Cardiovascular disease / atherosclerosis	Copper or silver wire arterioles, retinal emboli, Hollenhorst plaques	Stroke, transient ischemic attack (TIA), other thromboembolic events, cardiovascular events
Central Retinal Vein Occlusion	Widespread retinal Hb, dilated and tortuous retinal veins, CWS, macular oedema	Permanent vision loss (potentially severe), neovascular glaucoma (painful and blinding)
Central Retinal Artery Occlusion	Retinal whitening, cherry-red spot at the macula, box-carring of retinal vessels	Irreversible vision loss (potentially profound), risk of associated embolic stroke, need for urgent intervention to attempt reperfusion
Raised intracranial pressure	Blurred optic disc margins, obscuration of overlying vessels, venous engorgement with loss of venous pulsations	Risk of missing serious intracranial pathology (tumour, venous thrombosis) resulting in permanent vision loss, neurological deficits and death
Systemic Infections	HIV retinopathy (CWS, retinal Hb), CMV retinitis (white retinal lesions with Hb), Toxoplasmosis (focal necrotizing retinitis)	Progression of systemic infection, vision loss, opportunistic infections in other organs, increased morbidity and mortality
Blood Disorders	Sickle cell retinopathy (salmon-patch Hb, neovascularization) Leukemia (retinal Hb, CWS, roth spots)	Complications related to the underlying blood disorder (e.g., vaso-occlusive crisis in sickle cell disease, increased bleeding risk in leukemia)
Connective Tissue Disorders	Lupus retinopathy (CWS, retinal Hb Scleroderma (retinal vascular occlusions)	Systemic complications of the underlying connective tissue disease (e.g., kidney involvement in lupus, pulmonary hypertension in scleroderma)
Age-Related Macular Degeneration	Drusen, geographic atrophy, choroidal neovascularization	Progressive vision loss, central vision loss impacting reading and driving, decreased quality of life
Giant Cell Arteritis	Pallid optic disc oedema,CWS, possible central retinal artery occlusion	Irreversible vision loss, stroke, systemic complications (aortic aneurysm, other vascular events), increased morbidity and mortality if untreated
Intracerebral Mass	Papilledema (often bilateral), possible cranial nerve palsies	Permanent vision loss, neurological deficits, hydrocephalus, compression of vital structures in the brainstem leading to respiratory failure, coma, and death
Retinal Melanoma	Elevated pigmented or non-pigmented mass, subretinal fluid, possible associated vitreous Hb	Metastasis to other parts of the body (liver, lung, bone), potentially leading to death. Early detection and treatment improve prognosis

In a prospective, crossover management impact study, Dunn et al [10] were the first to demonstrate a clear impact on EP decision-making processes with the inclusion of NMFP images in patient assessment. They determined that implementing NMFP resulted in a meaningful change to management for 39% of 133 ER patients. Of these patients with a change in management after NMFP was shown to EPs, 34 changes (65%) involved escalation of management with additional investigations, referrals, or hospital admissions due to abnormal fundus findings; while 18 (35%) were de-escalations of management with less investigation or intervention made on the basis of normal fundus findings (see case vignette).

Biousse et al. published interim findings of an ongoing study evaluating the short-term impact of NMFP implemented in a busy metropolitan quaternary ED setting [43]. This is a prospective investigation, accumulating NMFP images from patients presenting to the ER with visual complaints, headaches, neurologic symptoms, “papilledema”, or hypertensive crisis. In its first four months, 385 patients had NMFP images taken. The photographs were interpreted via a telemedicine model, with remote ophthalmology review of images. NMFP images were found to accelerate the management and disposition of patients with acute pathology in 56.3% of enrolled patients, including 13 cases of acute central retinal arterial occlusions, 5 of giant cell arteritis, and 44 of papilledema. This ongoing study emphasises the ER workflow benefits offered by NMFP screening protocols—reducing length of stay, increasing the speed of diagnosis, and expediting relevant management for vision or life-threatening pathology. This study is continuing, with data to be collected for a total of 9 months.

**Fig. 2:**
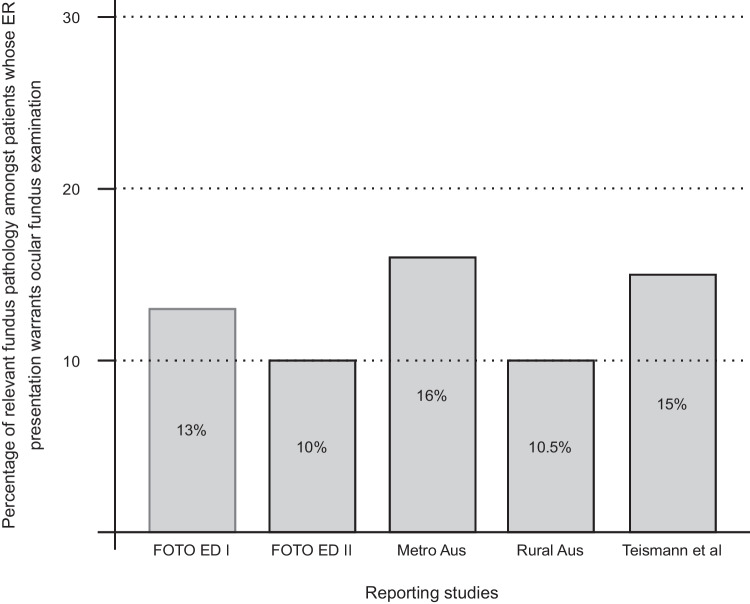
Period prevalence of fundoscopic pathology in patients whose presentation to the emergency room across studies reporting comparable statistics including the FOTO ED studies (phases I and II), Dunn et al.’s studies in both metropolitan and regional studies, and Teismann et al.’s study in a major US-based ER [10–12, 32, 41]: ‘FOTO ED’ = The fundus photography vs ophthalmoscopy trial outcomes in the emergency department sequential studies; ‘Aus’ = Australia; ‘Metro’ = Metropolitan

The prevalence of fundoscopic pathology amongst relevant ER presentations seems similar across studies in the US, as well as in Australian metropolitan and rural ERs (see Fig. 2). This fundal pathology has clinical prognostic importance, as Bruce et al. found that abnormal fundus photographs on ER presentation were associated with a two-fold increase in subsequent hospital admission and four-fold increased rate of all-cause mortality [44].

## Telemedicine and NMFP: Enhancing ER Consultations

Telemedicine is the use of a structured clinical interaction, conducted whilst physically separated from the patient using various technological platforms in order to provide interview or examination information to a consulting provider as well as diagnostic or treatment information to a patient. The potential benefits of integrating telemedicine into ER settings include improved access to specialist care. This can lead to faster diagnosis and more appropriate treatment plans for patients. Additionally, telemedicine may reduce length of stay by facilitating quicker decision-making processes and improving resource allocation within the ER. There are also limitations associated with implementing telemedicine. Adequate infrastructure is essential for successful integration; this includes reliable internet connections and suitable hardware, software, and IT support. Data security concerns must also be addressed to protect sensitive medical data transmitted during consultations. Furthermore, legal and ethical considerations such as practitioner licensing requirements across jurisdictions need careful consideration and management.

The demonstrated feasibility of NMFP in emergency room settings, with its ease of use, time efficiency, and high-quality image capture, combined with the issue of EP interpretation shortfalls, lends itself to integration with telemedicine applications, offering potential for remote expert consultation and improved patient care.

There are multiple models of ophthalmic care that can be provided, which fall into two major categories pertinent to the ER setting: synchronous, asynchronous, and hybrid of these two categories [45]. Synchronous telemedicine consists of real-time video/telephone clinical interactions, whereas asynchronous telemedicine uses store and forward review to specialty ophthalmology of images, video, or other clinical information without a real-time in-person interaction [46]. Telemedicine has found many applications within the ER, particularly in the field of stroke [47], and there is significant scope to expand this in the field of ophthalmology.

To the authors knowledge, no formal assessment of synchronous telemedicine using NMFP has been conducted in the ER. The static images captured during NMFP examination lends itself to an asynchronous model of telemedicine review and these asynchronous models of care may be a more feasible approach for NMFP implementation in the ER. Asynchronous telemedicine opens the possibility of remote review to a larger pool of ophthalmologists who would otherwise be too busy for synchronous telemedicine. One study used a telemedicine NMFP image review, in which images taken from a rural Australian ER were reviewed within 48 h by the on-call ophthalmology registrar, and subsequently the consultant ophthalmologist unless deemed urgent by the EP [10]. In this process, there were several patients with ocular pathology identified who then had a streamlined outpatient ophthalmology follow-up. Whilst not a primary outcome of this study, this asynchronous telemedical care model was safe, and improved patient outcomes. The majority of studies included in this review used similar processes, with formal review via off-site consultant ophthalmology, validating this asynchronous approach as feasible, appropriate, and safe [10–12, 31, 34]. Regardless of the mode used, synchronous or asynchronous,, patients will require pathways for in-person evaluation when necessary; thus any implementation requires forward planning to ensure adequate referral networks are established beforehand [10–12, 31, 34].

## Expert Eyes

When comparing the diagnostic accuracy of NMFP use by EPs to that of experts such as ophthalmologists, and neuro-ophthalmologists, the literature reveals some notable differences. Whilst many articles look at NMFP in ER and compare EPs interpreting ability to experts, they do not explicitly compare expert consultant review of NMFP imaging to other standards [9, 11, 12, 32]. Bursztyn et al. demonstrated that consultant ophthalmologist review of handheld NMFP images can achieve sensitivities for papilloedema between 72–92% when compared to clinical examination [35]. In a systematic review, the sensitivity and specificity of the variety of handheld NMFP devices was analysed [48], it found across all ocular pathology, handheld NMFP devices with expert image review facilitated a sensitivity of 85% and specificity of 91% when compared to expert examination using a non-NMFP gold standard. The disparity in diagnostic accuracy between EPs and experts, the latter achieving consistently higher performance due to their expertise have led to the general consensus in the literature that NMFP offer acceptable levels of diagnostic accuracy when reviewed by ophthalmologists who are supporting the EPs interpretation [11, 31, 46].

A gap in the research exists in evaluating the efficacy of NMFP implementation in ER settings where expert review is unavailable, even via telemedicine. This could include situations such as rural and remote location, or environments where ophthalmology specialists are reluctant to participate in the process. Further investigation is needed to determine whether NMFP remains a valuable tool in these more ambiguous contexts.

## NMFP Beyond the ER

The advantages of NMFP extend beyond its applications in the ER, it is already a valuable tool used in various clinical settings. It's utility has been established in DR screening [49], neurology and outpatient medicine [14, 50, 51], and general practice [52].

In neurology, the FUNDUS study discussed earlier relating to its comparison of devices, is a prospective cross-sectional surveillance and diagnostic accuracy study of adult neurology inpatients [14]. It highlighted NMFP's potential finding that 14% (95%

CI 7.3–23.8%) of the 79 enrolled patients had neurologically significant fundus.

pathology. Notably, DO was performed by the neurology team in only 6.6% of cases, and all abnormalities were missed.

NMFP is increasingly used in DR screening programs, mirroring the feasibility and efficiency seen in ER applications [53, 54]. The integration of NMFP into these screening programs has shown great promise, particularly in nurse-led initiatives. Fernández-Gutiérrez et al. [42] found that primary care nurses performing fundus photography for DR screening had adequate knowledge of the condition, and good agreement with ophthalmologists regarding screening decisions, reinforcing the viability of nurse-led screening programs. In their study, the agreement rate for screening results between nurses and ophthalmologists was 90%. These efforts have improved access to screening and early detection of DR**.**

The Diabetic Retinopathy Screening at the Point of Care (DR SPOC) initiative [55] exemplifies non physician led approach by integrating portable technologies within existing services in high-risk tertiary clinics. A high prevalence of previously undiagnosed DR and vision-threatening DR was found among patients attending foot ulcer and integrated care diabetes clinics in Western Sydney. In the 273 patients, 39.6% had some DR, while 15.8% had vision threatening DR, of whom 59.3% and 62.8% were previously undiagnosed, respectively [55]. While NMFP alone had limited sensitivity in their study, the DR SPOC initiative highlights the potential of point-of-care DR screening to enhance detection, particularly when combined with mydriasis. It was emphasised that portable, mydriatic photography demonstrates potential as an easily accessible model of care targeted for high-risk populations.

While NMFP has proven to change outcomes in a multitude of clinical settings, further research is needed to explore its potential in primary care settings such as general practice. Additionally, investigating the collaborative implications of its widespread use in optometry could provide insights into how NMFP has expanded access to eye care for patients with diabetes and other conditions.

## An Eye to the Future: Transforming Emergency Care

New frontiers with artificial intelligence (AI) systems designed to review and report medical images are now being applied to NMFP image databases to aid rapid NMFP image assessment. These AI systems often utilise deep learning algorithms, particularly convolutional neural networks which have shown remarkable performance in image recognition [56]. They are especially effective in medical image analysis, as they can automatically learn and extract necessary features for efficient image interpretation [57]. Acknowledging the limitations of AI based systems is crucial. Bias in training data can lead to skewed results, particularly if the data lacks diversity or is not a true representation of a patient population. Additionally the lack of transparency in AI decision making processes, often referred to as the ‘black box’ problem, can make it difficult for clinicians to understand and trust AI outputs [58].

Biousse et al. [59] recently published early data from their brain and optic nerve study artificial intelligence (BONSAI) deep learning system, applied to 1608 NMFP images taken from 828 patients obtained during the FOTO-ED studies. The preliminary data shows that the BONSAI deep learning system reliably identified papilloedema from normal and others with sensitivity of 84% (specificity 98.9%), and abnormal optic discs from normal with sensitivity 75.6% (specificity 89.6%) when compared to reference.

This highlights the potential for deep learning systems to augment NMFP image interpretation within a screening system, and potentially help in the decision-making processes which further benefit patient and departmental outcomes. The United States Food and Drug Administration recommended a threshold of 85% sensitivity, and 82.5% specificity for the approval of AI screening tools for DR [60]. Applied to ER-captured NMFP images, it is clear such diagnostic aids are becoming increasingly feasible for implementation.

AI diagnostic support should reduce human diagnostic error, and overcome some barriers to broader implementation of NMFP in the ER, however the need remains for clinicians to upskill in fundus interpretation to crosscheck AI outputs, and to integrate fundus findings into patient-specific clinical management. The ideal mix of clinician education, telemedicine and AI required to optimise clinical integration of NMFP in the ER will be an evolving research and policy question as these technologies mature. Optical coherence tomography (OCT) is another OF assessment tool, which has potential to be combined with NMFP screening in the ER to offer improved diagnostic acumen for retinal pathology [61]. However this is an early field and health-economic research is needed to clarify its potential.

## Conclusions

Non mydriatic fundus photography is a superior alternative to direct ophthalmoscopy for ocular fundus examination by the non-ophthalmic clinician, offering consistent and improved detection of pathology. There is now a wide array of non-mydriatic technologies available, all offering advantages to direct ophthalmoscopy in the emergency room. Limited emergency physician diagnostic accuracy for fundus pathology means ophthalmological oversight is still optimal in the diagnostic process, however handheld non-mydriatic cameras also lend themselves to integrated, asynchronous telemedicine models of care. Non mydriatic fundus photography screening protocols have been shown to impact management decision-making, improving patient outcomes. Ongoing research and implementation efforts are required to improve the uptake of non-mydriatic fundus photography screening protocols in the emergency room, help educate emergency physicians in fundus interpretation, and integrate emerging possibilities from artificial intelligence-driven diagnostic tools for non mydriatic fundus photography screening protocols in the emergency room.

## Clinical Vignette

**Fig. 3:**
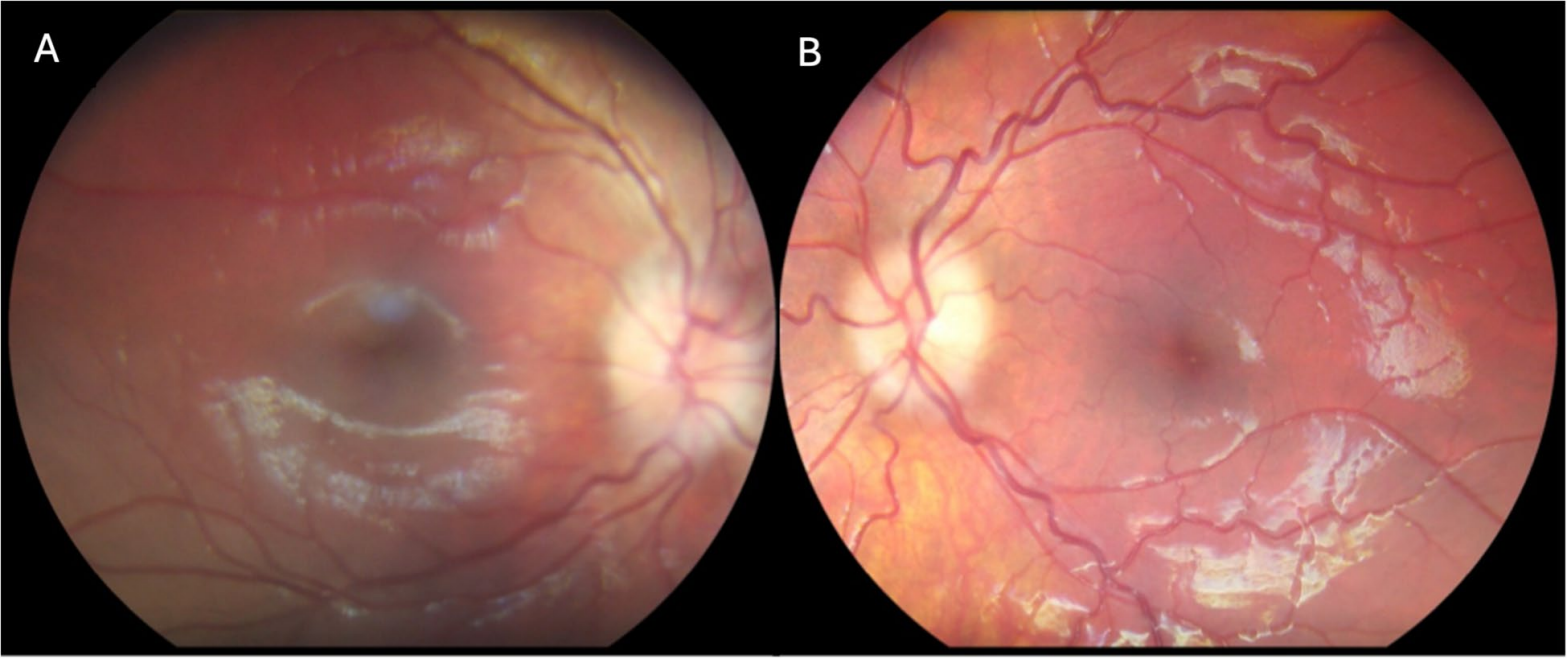
Macula-centred non mydriatic fundus images taken from the paediatric patient on presentation to the emergency room, **A** right eye; **B** left eye

During the data-collection phase of Dunn et al. [10] study on the impact of NMFP screening on ER-management of patients, a 6 year old patient (ultimately excluded from analysed data due to age-related exclusion criteria) presented having had several months of intermittent headaches, and occasional vomiting. She had presented to local ER services one month into these symptoms, and was sent home without a fundoscopic examination, with symptoms ascribed to a recent viral illness. On subsequent presentation to ER with now new blurred vision in her left eye, fundus images were taken using the handheld non-mydriatic fundus camera, revealing bilateral moderate to severe papilloedema (See figure). This led to an urgent paediatrics consult and MRI brain, showing a large brainstem mass with midline shift, for which the patient was urgently transferred for neurosurgical resection of the mass. This case epitomizes the important pathology that can be missed in the absence of appropriate ocular fundus screening/examination protocols in the ER setting and highlights the important gap in ocular assessment that NMFP screening can fill (Fig. 3).

## Key References

- Biousse, V. et al*.* Application of a Deep Learning System to Detect Papilledema on Nonmydriatic Ocular Fundus Photographs in an Emergency Department. *Am. J. Ophthalmol.* **261**, 199–207 (2024).
  - Retrospective analysis shows AI assisting in ER-based NMFP image review, supporting further exploration of its broader implementation.
- Bruce, B.B., et al. 2013. Nonmydriatic ocular fundus photography for the detection of papilledema in the emergency department. *Annals of Emergency Medicine*, 62(1), pp.1–9.
  - Prospective observational study assessed the diagnostic accuracy of NMFP in its ER application, highlighting critical improvements compared to direct ophthalmoscopy
- Bruce, B.B., et al. 2011. Nonmydriatic ocular fundus photography in the emergency department. *New England Journal of Medicine*, 364(4), pp.387–389.
  - First landmark investigation to apply relatively new NMFP technology in the ER setting, highlighting its feasibility in this environment.
- Dunn, H. et al. 2022. Integration of non-mydriatic fundus photography into emergency workflow: Impact on physician decision-making and diagnostic accuracy. *Emergency Medicine Australasia*, 34(3), pp.300–306.
  - Prospective study builds on FOTO-ED findings, showing that integrating NMFP images into ER workflow improves diagnostic accuracy and enhances decision-making
- Dunn, H. et al. 2021. Detection of ocular fundus pathology in Australian emergency departments using non-mydriatic fundus photography. *Emergency Medicine Australasia*, 33(2), pp.214–220.
  - Validated previous FOTO-ED studies by highlighting the prevalence of pathology in Australian metropolitan ERs, and demonstrating the feasibility and diagnostic accuracy.
- He, M., Kaushik, S., Ananda, A. and Yeo, T., 2022. Prevalence of ocular pathology among neurology inpatients using non-mydriatic fundus photography. *Internal Medicine Journal*, 52(4), pp.512–518.
  - Prospective study found prevalence of ocular pathology among neurology inpatients in an Australian tertiary hospital, underscoring the importance of fundus screening in ER patients
- Teismann, N.A., Harrison, S., Thorson, K., Shah, K.H. and Biousse, V., 2019. Validation of nonmydriatic fundus photography in a contemporary emergency department setting. *The Journal of Emergency Medicine*, 56(5), pp.592–598.
  - Validated previously demonstrated ER-feasibility of NMFP images in a more contemporary setting

## Data Availability

No datasets were generated or analysed during the current study.
